# *Free and Easy Wanderer* Ameliorates Intestinal Bloating‐Dependent Avoidance Behavior of *Caenorhabditis elegans* Through Gut‐Germline‐Neural Signaling

**DOI:** 10.1111/cns.70291

**Published:** 2025-02-26

**Authors:** Siyi Lin, Huangjie Fu, Hanxiao Wang, Yingying Xu, Yu Zhao, Shiyu Du, Jiale Wei, Ping Qiu, Senlin Shi, Changyu Li, Thomas Efferth, Chunlan Hong

**Affiliations:** ^1^ School of Pharmaceutical Sciences Zhejiang Chinese Medical University Hangzhou China; ^2^ School of Basic Medical Sciences Zhejiang Chinese Medical University Hangzhou China; ^3^ Department of Pharmaceutical Biology, Institute of Pharmaceutical and Biomedical Sciences Johannes Gutenberg University Mainz Germany

**Keywords:** *free and easy wanderer*, gut‐germline‐neural signaling, H4K8ac, intestinal bloating

## Abstract

**Purpose:**

The aim of this study was to investigate the protective effect of *Free and Easy Wanderer* (FAEW) on the avoidance behavior induced by feeding Heat‐Killed *Escherichia coli* , and to elucidate the underlying mechanisms.

**Methods:**

Initially, the effects of FAEW on avoidance behavior, survival, neuroendocrine signaling gene expression, and intestinal bloating were examined. The impact of FAEW on gut‐germline‐neural signaling was assessed by monitoring H4K8ac expression and the avoidance behavior of *par‐5* RNAi animals and *glp‐1(e2141)* mutants. RNA‐sequencing was conducted to analyze potential signaling pathways. Finally, avoidance behavior was examined using *daf‐16(mu86)* mutants and the rescued animals.

**Results:**

FAEW delayed avoidance behavior. FAEW significantly downregulated gene expression in the neuroendocrine signaling pathway and alleviated intestinal bloating of *C. elegans* . The levels of H4K8ac and *par‐5* in the germline decreased significantly with FAEW's treatment, and FAEW failed to affect the avoidance behavior of *par‐5* RNAi animals and *glp‐1(e2141)* mutants. FAEW's effect on avoidance behavior diminished in *daf‐16(mu86)* mutants but was restored in *daf‐16* rescued animals. FAEW has been observed to restore *daf‐16* levels.

**Conclusion:**

FAEW protects against avoidance behavior of *C. elegans* through downregulating H4K8ac protein expression and activating DAF‐16. This study provides crucial experimental evidence supporting FAEW as a promising candidate for protecting against avoidance behavior associated with PTSD.

## Graphical abstract

Using *C. elegans* as a model organism, we scrutinized the effects of FAEW on pathogen avoidance behaviors via the gut‐germline‐neural signaling. Demonstrating for the first time that FAEW prevents avoidance behavior through gut‐germline‐neural signaling, inhibition of H4K8 acetylation, and activation of DAF‐16.

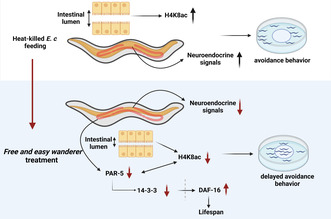

**Abbreviations**

*C. elegans*

:
*Caenorhabditis elegans*

DEGs: differentially expressed genes
*E. c*
:
*Escherichia coli*

FAEW: *Free and easy wanderer*
FXT: fluoxetine
GEO: Gene expression omnibus
GO: Genome ontology
HK *E. c*
: heat‐killed *Escherichia coli*

IPTG: isopropyl β‐D‐1‐thiogalactopyranoside
KEGG: Kyoto encyclopedia of genes and genomes
LB: Luria‐Bertani
NGM: nematode growth medium
*P. a*
:
*Pseudomonas aeruginosa*

PTSD: posttraumatic stress disorder
RNAi: RNA interference
UPLC–Q‐TOF/MS: Ultra‐high performance liquid chromatography with quadrupole time‐of‐flight mass spectrometry

## 1 Introduction

PTSD is an enduring and delayed mental condition characterized by a constellation of symptoms, including persistent avoidance, heightened awareness and alertness, negative cognitive processes and emotions, as well as intrusive experiences. The enduring nature of PTSD significantly impacts both the physical and mental well‐being of individuals. Unfortunately, effective therapeutic strategies for combating PTSD are currently lacking in clinics. Neurosteroids and neurotrophic factors have potential as biomarkers for major depression and PTSD [1]. Selective brain steroidogenic stimulants ameliorate behavioral deficits by normalizing allopregnanolone biosynthesis in a mouse model associated with PTSD [2]. Furthermore, the complex link between the central nervous system and the enteric nervous system is well established [3, 4]. Changes in the gut microbiota and associated PTSD symptoms have been explored in clinical studies in populations of individuals affected by PTSD [5, 6]. A gram‐positive immunomodulatory strain of the probiotic *lactobacillus rhamnosus* was used in a clinical trial (NCT04150380) to treat veterans with PTSD [7].

*Caenorhabditis elegans* ( *C. elegans* ) is a non‐parasitic roundworm that predominantly consumes *Escherichia coli* (*E. coli*). Within its system, a diverse array of neurotransmitters, such as dopamine, acetylcholine, and 5‐hydroxytryptamine, are present. The synthesis, storage, and metabolism processes of these neurotransmitters closely mirror those found in mammals [8], and remain conserved in certain neuroendocrine signaling pathways with mammals [9]. This intriguing parallel underscores the potential of *C. elegans* as a valuable model organism for drug discovery in the field of neuropsychiatric diseases. Additionally, it has been demonstrated that during the L1 larval stage of *C. elegans* , exposure to *Pseudomonas aeruginosa* (*P. a*) results in adult worms exhibiting an early avoidance behavior towards *P. a* [10]. During the L4 larval stage, whether exposed to *P. a* or induced with intestinal distension by feeding on HK *E. c*, the next generation of *C. elegans* exhibits early avoidance behavior towards *P. a* [11]. Thus, *C. elegans* exhibits avoidance behavior in harmful situations, similar to avoidant‐type symptoms in PTSD patients.

However, the specificity of PTSD pathogenesis, including the overlap of symptoms with other psychiatric disorders, complicates the construction of a well‐developed model of PTSD. In this regard, we used *C. elegans* , a simple model organism, to simulate the avoidance symptoms and delve into the subsequent mechanisms of avoidance behavior. The traditional Chinese medicine compound FAEW was selected for intervention. By examining the avoidance behavior, intestinal distension, the gene expression of neuropeptide, the level of H4K8ac in the germline, that is, the intestinal‐germline‐neural axis, as well as DAF‐16 signaling using gene silencing and mutant animals, the effects of FAEW on the mechanisms of avoidance behavior are elucidated.

## 2 Materials and Methods

### 2.1 Bacterial Strains

The following bacterial strains were used: *E. c*. and *P. a*. Bacterial cultures were grown in Luria‐Bertani (LB) broth at 37°C.

### 2.2 Nematode Strains and Growth Conditions

*C. elegans* hermaphrodites were maintained on *E. c* at 20°C, except HH142 *fer‐1 (b232)* and CB4037 *glp‐1(e2141)* strains that were maintained at 15°C. Bristol Wild‐type N2 was used as the wild‐type control. HH142 *fer‐1(b232)*, CB4037 *glp‐1(e2141)*, CF1038 *daf‐16*(*mu86*), and CF1139 *daf‐16(mu86) I; muIs61* strains were obtained from the *Caenorhabditis elegans* Genetics Center (University of Minnesota, Minneapolis).

### 2.3 Treatment of *Free and Easy Wanderer* and Fluoxetine

The traditional Chinese herb formula FAEW was purchased from Guangdong Zhengyuan Molecular Chinese Medicine Co. A highly concentrated FAEW stock solution was prepared by dissolving molecular weight FAEW in distilled water and then filtering through a 0.22 μm membrane. Long‐term storage was at −4°C. A gradient diluted FAEW plate was prepared by mixing FAEW stock solution into the medium. Under the condition that the growth of *E. c* was not affected, high and low doses were selected to carry out the experiment. Fluoxetine hydrochloride was purchased from Shanghai Eon Chemical Technology Co Ltd. (Shanghai, China). It was filtered through a 0.22 μm filter membrane and added to the plate according to the experimental method described above.

### 2.4 Bacterial Lawn Avoidance Assay

The bacterial lawns were generated by isolating individual colonies of *P. a*, which were then cultured in 2 μL of LB medium and incubated at 37°C for 12 h on a shaker. Subsequently, a 20 μL aliquot of the culture was applied to the center of a 3.5‐cm modified nematode growth medium (NGM) plate and incubated at 37°C for an additional 12 h. For each experimental condition, 20 synchronized hermaphroditic animals were utilized. The transfer of these animals into the bacterial lawn was followed by counting the number of animals present both on and off the lawn at specified time points during each experiment. The experiments were predominantly conducted at 25°C. The calculation of percent occupancy was determined by the formula (N_on‐lawn_/N_total_) × 100.

### 2.5 RNA Interference (RNAi)

RNAi experiments were conducted according to established protocols detailed in prior studies [12]. In brief, *E. c* strains carrying the relevant vectors were cultured in LB broth supplemented with ampicillin (100 μg/mL) at 37°C overnight. The cultured bacteria were then plated onto NGM plates containing 100 μg/mL ampicillin and 3 mM isopropyl β‐D‐1‐thiogalactopyranoside (IPTG) for RNAi induction (referred to as RNAi plates). Following an overnight incubation at 37°C, RNAi‐expressing bacterial colonies were generated. Subsequently, synchronized animals at the L4 developmental stage were transferred onto the RNAi plates and cultured for 24 h at 25°C. It is important to note that all RNAi clones were obtained from the Ahringer RNAi library.

### 2.6 Pharyngeal Pumping Assay

Wild‐type N2 animals grown on HK *E. c* for 24 h were used for the pharyngeal pumping assay with animals grown on live *E. c* as controls. The number of contractions of the terminal bulb was counted over 1 min. A contraction was defined as the backward movement of the grinder in the terminal bulb of the pharynx. The pumping rates for 20 worms were recorded for each condition.

### 2.7 Defecation Cycle Assay

Wild‐type N2 animals grown on HK *E. c* for 24 h were used for the defecation assay, with animals grown on live *E. c* as controls. The defecation cycle length was scored by assessing the time between expulsions. Five cycles each were measured for six different animals per condition.

### 2.8 Killing Assay on *P. a*

The *C. elegans* killing assay was conducted using wild‐type *P. a* lawn, which was incubated at 37°C for 12 h. 50 μL of an overnight culture of *P. a* grown at 37°C was evenly spread on the entire surface of 3.5‐cm‐diameter SK plates. The plates were then incubated at 37°C for 12 h before seeding with synchronized young gravid adult hermaphroditic animals. The killing assays were conducted at 25°C, with live animals transferred daily to fresh plates. Animals were assessed at specified time points and considered dead if unresponsive to touch.

### 2.9 Lifespan Assay

The animals were transferred onto new plates containing *E. c*. These plates were supplemented with streptomycin (100 mg/mL), kanamycin (50 mg/mL), and nystatin (10 mg/mL) to prevent contamination. A 350 μL drop of the HK bacteria was plated on a 6 cm plate and incubated at 25°C. Survival assessments were conducted at specified times, with live animals transferred to fresh plates as needed. Each experiment was replicated three times, with 50 worms in each group and triplicate sets.

### 2.10 RNA Isolation and Quantitative Real‐Time PCR

The animals were then washed off the plates with M9 buffer three to four times and frozen in TRIzol (Accurate Biotechnology (Hunan) Co. Ltd. Chang Sha, China). Subsequently, 10 μg of total RNA was reverse transcribed with random primers using the Evo *M‐MLV* Reverse Transcription Kit (Accurate Biotechnology (Hunan) Co. Ltd. Chang Sha, China). Quantitative RT‐PCR was performed using SYBR Green Premix *Pro Taq* HS qPCR Kit (Accurate Biotechnology (Hunan) Co. Ltd. Chang Sha, China) on a BIO‐RAD CFX96 real‐time PCR machine in a 96‐well plate format, utilizing 5 μL in each reaction. The relative fold‐changes of the transcripts were calculated using the comparative CT (2^−ΔΔCT^) method and normalized to pan‐actin values. Data analysis was conducted using the CFX96 Touch Real‐Time PCR Detection System (BIO‐RED, US). Three technical replicates of samples were utilized, and the experiment was repeated three times for robustness and reliability. The primers used to measure the indicated genes were listed in Table S1.

### 2.11 Intestinal Bloating

The worms were collected and washed with M9 three times. The adult worms were paralyzed with 5 mM Levamisole hydrochloride and then mounted to a 2% agar pad. The anterior and posterior intestine regions were taken on a Positive fluorescence microscope (AXIO SCOPE.A1, Zeiss, Germany).

### 2.12 Identification of Composition of FAEW

A precisely measured dried sample of FAEW weighing 1 g was placed into a centrifuge tube. Subsequently, 10 mL of water was added to the tube, and the resulting solution was filtered. The analysis was performed using a Waters ACQUITY I‐Class Plus UPLC system from Waters, USA, featuring a TurboIonSpray source (AB SCIEX, USA) and a quadrupole time‐of‐flight mass spectrometer SCIEX X500R (AB SCIEX, USA). Both ESI positive‐ and negative‐ion scanning modes were employed in the study. The acquired UPLC–Q‐TOF/MS data were processed and interpreted using SCIEX OS software. Target compounds were identified utilizing the secondary database of the Traditional Chinese Medicine (TCM) MS/MS Library.

### 2.13 Whole Mount Fluorescent Immunohistochemistry

Synchronized young adult animals were exposed to either *P. a* or *E. c* for 24 h at 25°C. Subsequently, worms were washed with M9 and resuspended in a fixing solution (KCl, Tris–HCl (pH 7.4), NaCl, Na_2_EGTA, EDTA, spermidine HCl, PIPES (pH 7.4), Triton X‐100, methanol, formaldehyde) before being snap‐frozen in liquid nitrogen. The worms were fixed on ice for 4 h and briefly washed in T buffer (Tris–HCl (pH 7.4), EDTA, Triton X‐100) before a 15 min incubation in T buffer supplemented with β‐mercaptoethanol at 37°C. Following this, the worms were washed with borate buffer and then incubated in borate buffer containing DTT for 15 min, followed by H_2_O_2_ incubation for an additional 30 min. Subsequently, the worms were blocked in PBST containing 1% BSA for 30 min. They were then incubated overnight with anti‐H4K8ac antibody (1:100; ab15823, Abcam) and Alexa Fluor 594 secondary antibody (1:300; ab150080, Abcam). The worms were mounted on a microscope slide and observed using a stereofluorescence microscope (AXIO SCOPE.A1, Zeiss, Germany). The fluorescence intensity was quantified using Image J.

### 2.14 Statistical Analysis

Statistical analyses were performed with Prism 8 (GraphPad). A two‐tailed Student *t*‐test for independent samples was used to analyze the data. For comparing the means of more than two groups, a one‐way ANOVA with post hoc analysis was performed. The Kaplan‐Meier method was used to calculate the survival fractions, and statistical significance between survival curves was determined using the log‐rank test. All the experiments were repeated at least three times, and error bars represent the standard deviation. The data were judged to be statistically significant when *p* < 0.05, and the exact *p‐*values are listed in Table S2. “ns” indicates non‐significant. “*n*” represents the number of animals for each experiment.

## 3 Results

### 3.1 Composition Analysis of FAEW

**FIGURE 1:**
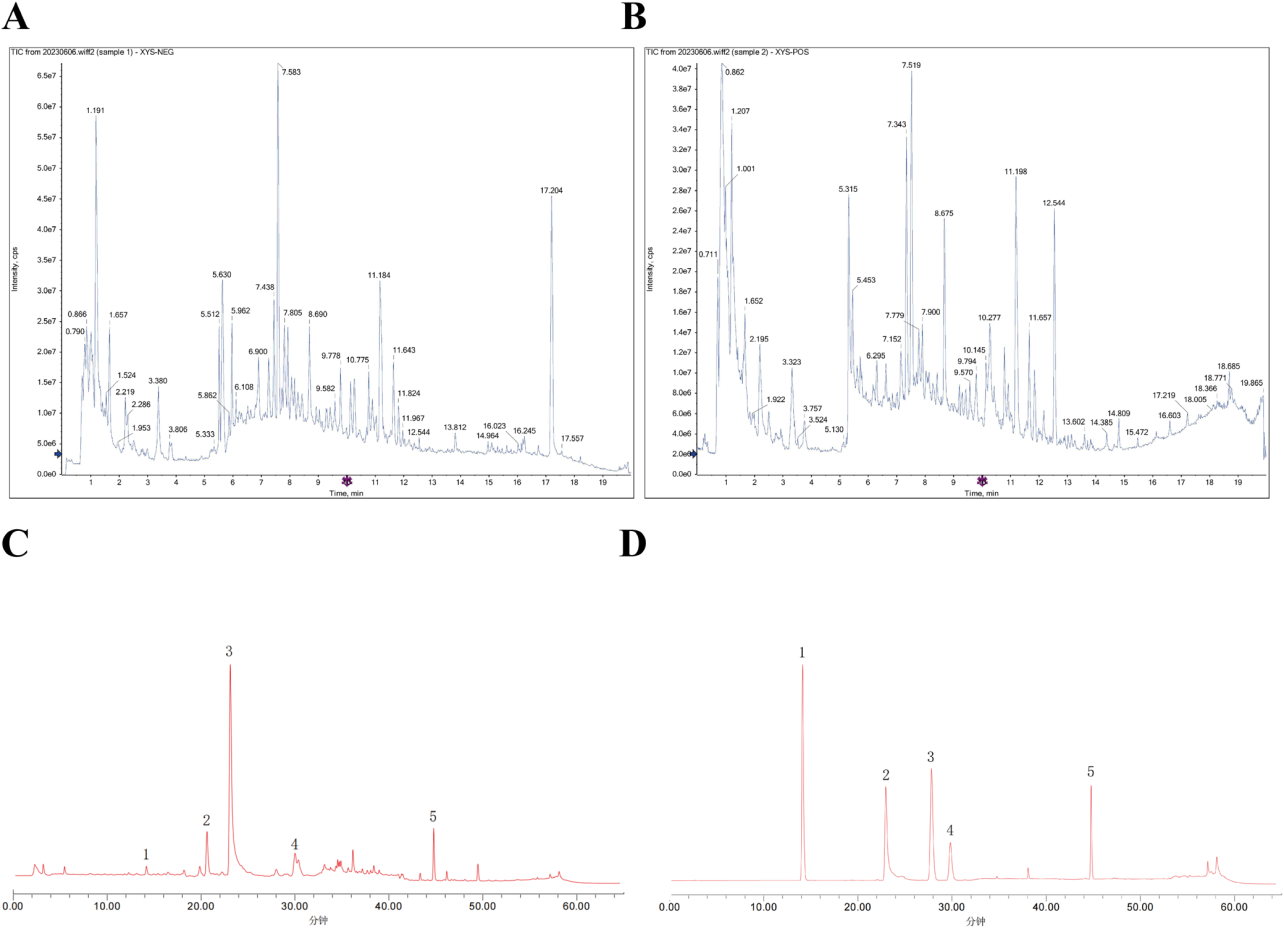
Five Components of FAEW extracts were identified by UPLC‐MS. (A) UPLC–HRMS base peak ion flow graph negative ion mode for FAEW. (B) UPLC–HRMS base peak ion flow graph positive ion mode for FAEW. (C) Chromatogram of the test material. (D) Chromatogram of mixed control. (1) Chlorogenic acid, (2) Paeoniflorin, (3) Ferulic acid, (4) Glycyrrhizin, (5) Glycyrrhizic acid.

The Chinese medicinal herb formula FAEW originates from “Taiping Huimin Heji Ju Fang,” representing a widely employed formula. This prescription comprises *Chaihu* (*Bupleurum chinensie DC*.), *Fulin* (*Poria cocos Schw. Wolf*), *Baizhu* (*Atractylodes Macrocephala*), *Baishao* (*Paeoniae Radix Alba*), *Danggui* (*Radix Angelicae Sinensis*), *Bohe* (*Mentha haplocalyx Briq*.), *Gancao* (*Radix Glycyrrhizae*), and *Shengjiang* (*Zingiber officinale Roscoe*). Clinically in China, FAEW is frequently employed in the treatment of neuropsychiatric disorders, gastrointestinal diseases and various other ailments [13]. To elucidate the primary compounds responsible for the pharmacological effects of FAEW, we initially used UPLC‐Q‐TOF/MS for the comprehensive analysis and identification of its components. As depicted in Figure 1A,B, a total of five major components of FAEW were successfully identified. Noteworthy constituents include chlorogenic acid, paeoniflorin, ferulic acid, glycyrrhizin, and glycyrrhetinic acid (Figure 1C,D).

### 3.2 FAEW Attenuates Intestinal Bloating‐Dependent Avoidance Behavior of *C. elegans*

In the defense against pathogens, *C. elegans* typically employs an avoidance response to enhance survival. Previous research by Alejandro Aballay's team revealed a connection between the avoidance behavior of *C. elegans* and the colonization of its gut by *P. a*. As *P. a* accumulates in the intestine, the intestinal lumen of *C. elegans* swells, leading to a significant upregulation of various immune genes such as *clec‐60* and *cpr‐2* [14]. Additionally, neuropeptide receptors, including *flp‐21* and *npr‐1*, are activated, triggering avoidance behavior. The onset of avoidance behavior in *C. elegans* is closely tied to immune function, with animals exhibiting lower immunity displaying earlier avoidance behavior.

**FIGURE 2:**
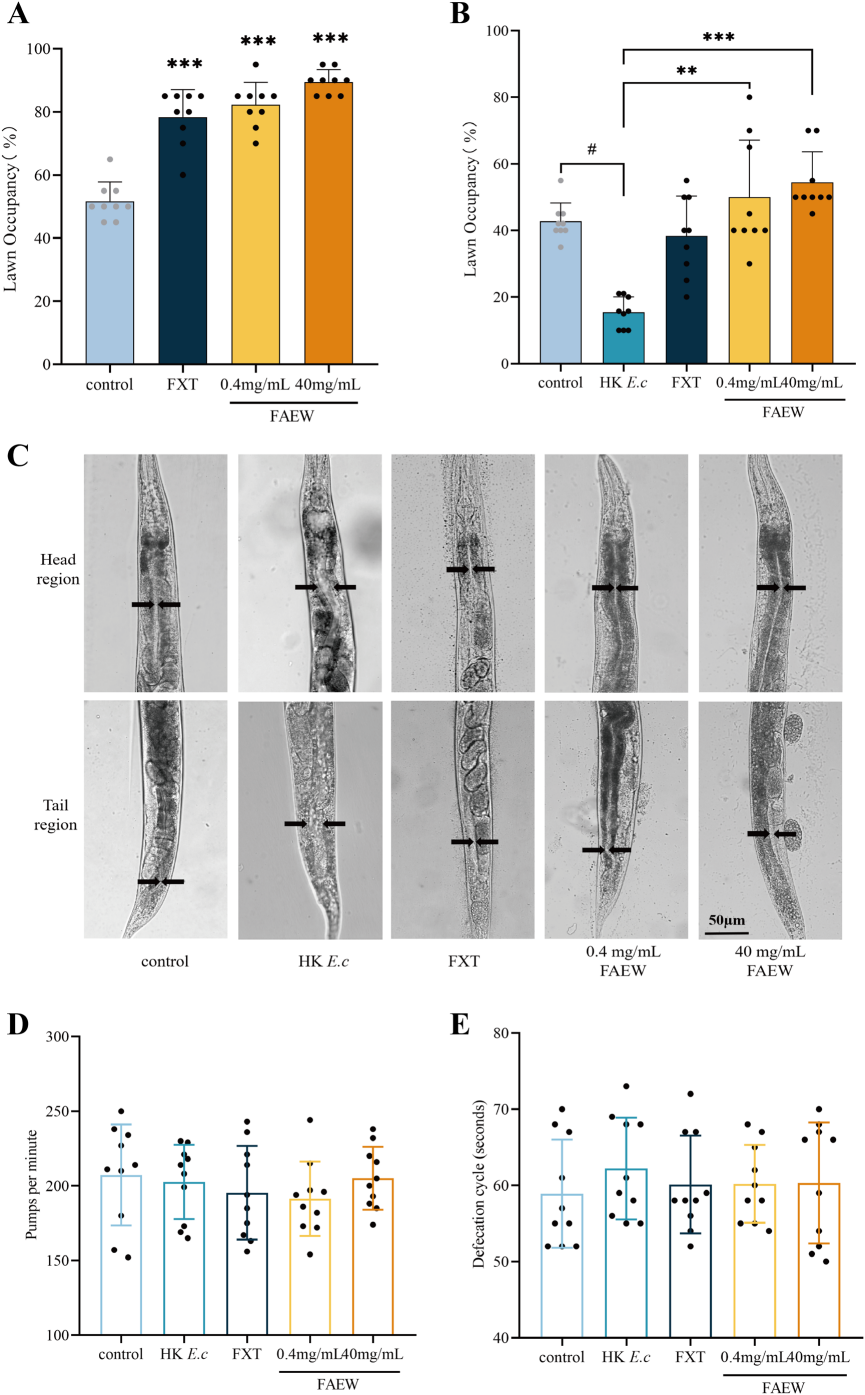
FAEW improves gut lumen expansion and delays avoidance behavior in *C. elegans* . (A) Lawn occupancy of wild‐type N2 animals exposed to *P. a* following FAEW and FXT interventions (FXT: 0.4 μM) (*n* = 20). (B) Lawn occupancy of *P. a* in animals with wild‐type N2 after FAEW and FXT treatment based on HK *E.* intervention (*n* = 20). (C) Representative photomicrographs of wild‐type N2 animals grown on *E. c* until the young adult stage, followed by incubation on HK *E. c* as well as on the administered plates for 24 h. Control animals were maintained on *E. c*. Arrows point to the border of the intestinal lumen. (D) Pharyngeal pumps per minute of Wild‐type N2 animals incubated on HK *E. c* for 24 h (*n* = 10). (E) Defecation cycle of Wild‐type N2 animals incubated on HK *E. c* for 24 h (*n* = 10). *Comparison with HK *E. c*; #Comparison with control; #*p* < 0.05; **p < 0.01; ***p < 0.001.

To assess the impact of FAEW on *C. elegans'* avoidance behavior, we used high (40 mg/mL) and low (0.4 mg/mL) dosages of FAEW to treat worms. Additionally, 0.4 μM fluoxetine (FXT) was used as a positive control. As shown in Figure 2A, a significant delay was observed in avoidance behavior upon the treatment of FAEW, suggesting that FAEW effectively postpones the onset of avoidance behavior in *C. elegans* . To investigate whether the immune system is involved in the modulation of avoidance behavior by FAEW, we conducted a survival assay to assess the impact of FAEW on immunity. Contrary to our expectations, FAEW did not enhance the innate immunity of *C. elegans* (Figure S1A). The relative mRNA levels of three PMK‐1/p38‐dependent immunity genes and the *clec‐60* gene were elevated after *P. a* infection; however, only F08G5.6 and *clec‐60* levels were relatively reduced after FAEW administration (Figure S1B–E). This finding suggests that multiple signaling pathways are involved in the innate immunity of *C. elegans* , and FAEW did not affect the innate immune response towards *P. a* infection.

Studies suggest that in the absence of defecation, motor program defects, or pathogenic microbial infections, *C. elegans* exhibited intestinal lumen distension and early avoidance. However, their pharyngeal pumping rate and defecation cycle remained unaltered compared to the normal diet group [15, 16, 17, 18]. To explore whether FAEW's impact on avoidance behavior is linked to gut lumen distension, we examined avoidance behavior and gut lumen distension in the presence of FAEW by feeding animals with HK *E. c*. The results showed that FAEW significantly prolonged *C. elegans* ' aversive behavior towards *P. a* after HK *E. c* feeding (Figure 2B) and improved intestinal lumen distension (Figure 2C). Nevertheless, the pharyngeal pumping rate and defecation cycle did not change (Figure 2D,E). This study suggests that FAEW improved intestinal lumen distension, consequently delaying avoidance behavior in *C. elegans* .

### 3.3 FAEW Modulates Neuroendocrine Signaling Pathway Gene Expression

*C. elegans* ' ability to sense pathogens is predominantly mediated by its nervous system, which perceives and responds to molecular inputs in the environment, akin to higher animals. The gene *tph‐1* plays a crucial role in the biosynthesis of 5‐hydroxytryptamine [19, 20]. Furthermore, it has been reported that the chemosensation of phenazine by pathogenic *P. a* activates DAF‐7/TGF‐β in ASJ neurons, impacting pathogen avoidance behavior [21]. Additionally, Intestinal infections and bloating, dependent on *P. a* virulence, regulate both pathogen avoidance and aversive learning through modulation of the DAF‐7/TGF‐β pathway and the NPR‐1/GPCR pathway, influencing chemotactic behavior and aversion learning [22]. Although the studies differ in some respects, they concur that the nervous system of *C. elegans* detects pathogen‐specific molecular signals, ultimately enabling the avoidance of pathogenic bacteria.

**FIGURE 3:**
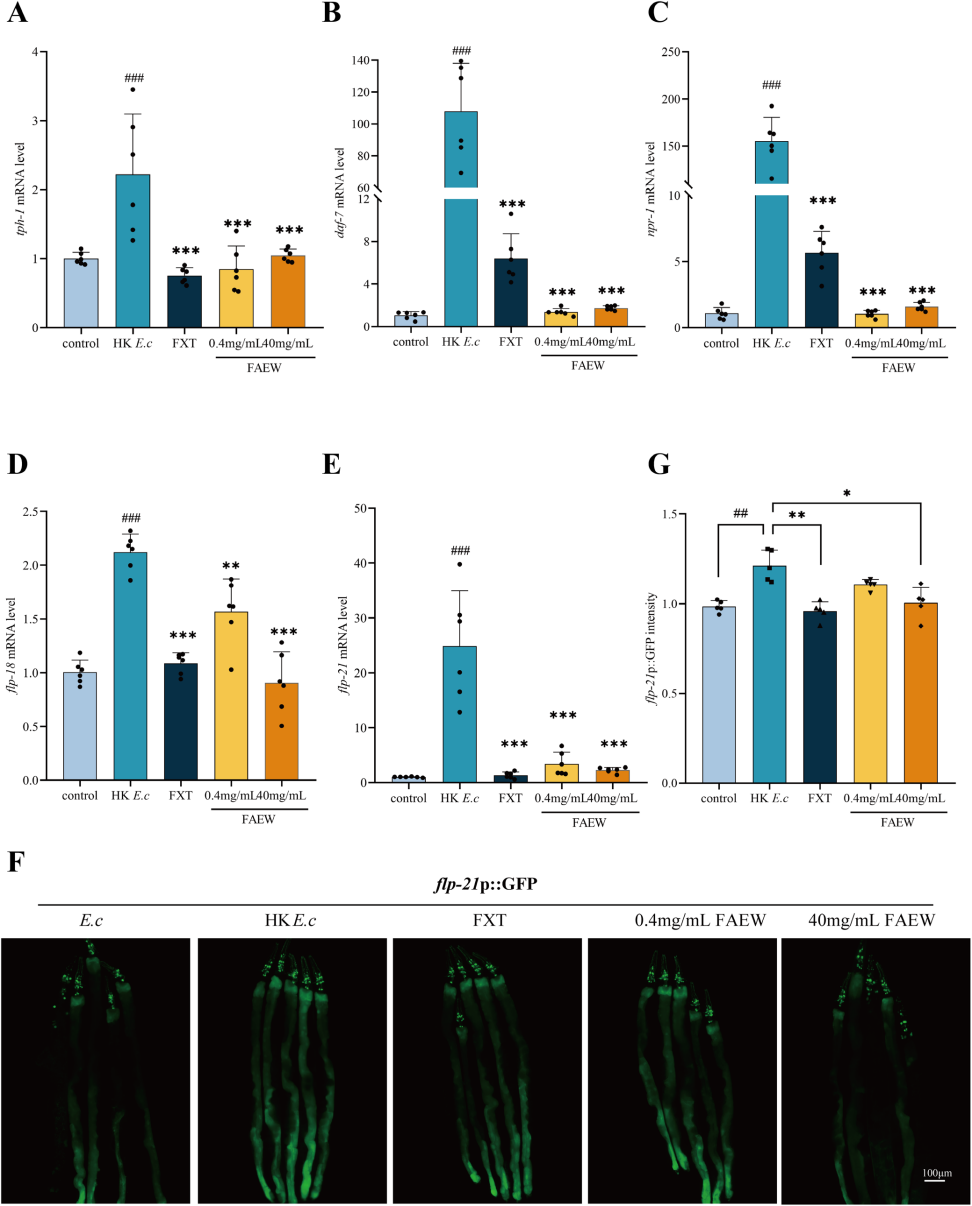
Effects of FAEW on neuroendocrine gene expression. (A‐E) Relative mRNA levels of *tph‐1* (A), *daf‐7* (B), *npr‐1* (C), *flp‐21* (D), and *flp‐18* (E) in *E. c* and animals grown on HK *E. c* bacteria following intervention with FAEW and FXT (*n* = 6). (F) Representative microscopic images of *flp‐21*p::GFP animals after intervention with FAEW and FXT. Scale bar, 100 μm. (G) Quantitative analysis of (E). The height of the column represents fold changes (*n* = 5). *Comparison with HK *E. c*; #Comparison with control; *p < 0.05; **, ##*p* < 0.01; ***, ###*p* < 0.001.

To explore the impact of FAEW on neuroendocrine signaling related to intestinal infections and bloating, we examined the gene expression of key components in the serotonin biosynthesis pathway (*tph‐1*) [23], the DAF‐7/TGF‐β pathway (*daf‐7*), and the NPR‐1/GPCR pathway (*npr‐1*, *flp‐18*, and *flp‐21*). As shown in Figure 3A–E, compared to the control group, the levels of *tph‐1*, *daf‐7*, *npr‐1*, *flp‐18*, and *flp‐21* in HK *E. c*‐fed animals significantly increased, while they were significantly decreased upon the treatment of FAEW. Additionally, the fluorescence of *flp‐21*p::GFP (NY2087) transgenic animals increased significantly after HK *E. c* feeding, and the effect diminished upon FAEW treatment, (Figure 3F,G). Taken together, these findings suggest that FAEW's effect in enhancing avoidance behavior of *C. elegans* is associated with a reduction in the expression of genes involved in multiple neuroendocrine signaling pathways at the molecular level.

### 3.4 FAEW Activates Multiple Pathways of *C. elegans*

**FIGURE 4:**
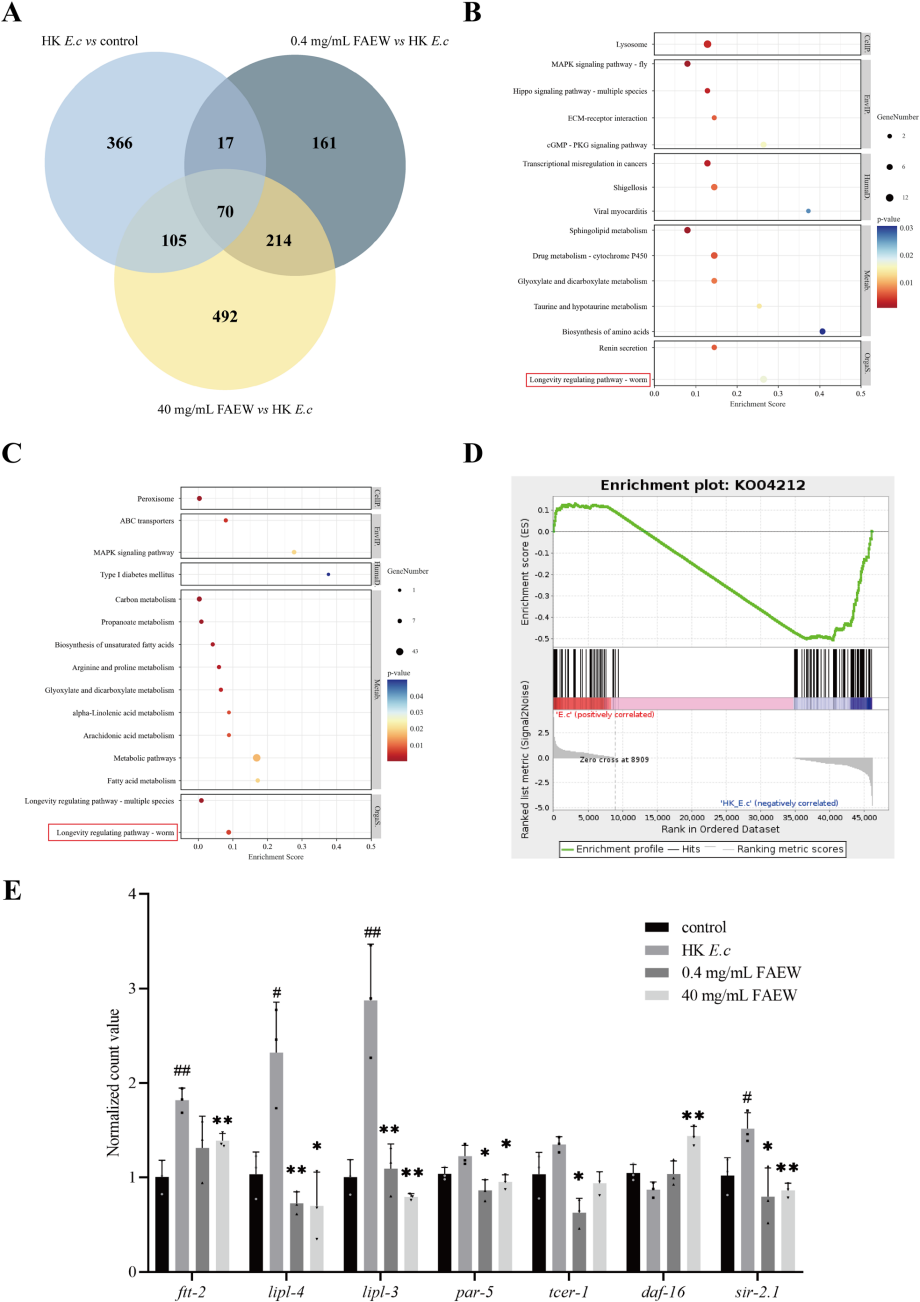
RNA‐seq analysis of the potential mechanisms underlying the delayed avoidance behavior in *C. elegans* induced by FAEW. (A) Venn diagrams illustrating the overlap of DEGs within each group. (B) KEGG enrichment analysis results of DGEs (HK *E. c* vs. control). (C) KEGG enrichment analysis results of DGEs (40 mg/mL FAEW vs. HK *E. c*). (D) GSEA enrichment analysis of the Longevity regulating pathway. (E) Longevity regulating pathway—worm relative expression levels of specific genes. *Comparison with HK *E. c*; #Comparison with control; *, #*p* < 0.05; **, ##*p* < 0.01.

To delve into the molecular mechanisms by which FAEW inhibits the avoidance behavior of *C. elegans* , we conducted RNA sequencing. The results showed that FAEW intervention resulted in the altered expression of multiple genes (Figure S2A–C). The Venn diagrams illustrating the differential genes in each group are shown in Figure 4A. Bioinformatics was performed to analyze the interactions between DEGs using the Ouyi Cloud platform, and KEGG pathway enrichment analysis indicated that DEGs were mainly associated with the involvement of the Longevity regulating pathway—worm (Figure 4B,C). Specific KEGG pathway maps are shown in Figure S2D,E. We are concerned that the *C. elegans* *ftt‐2* and *par‐5* genes act together on the 14‐3‐3 protein during germline ablation, and it has been documented that DAF‐16 is able to accumulate in the nucleus when 14‐3‐3 protein expression is reduced, which correlates with an increase in its transcription factor activity [24]. These results suggest that FAEW may affect *C. elegans* longevity and is complexly intertwined with avoidance behaviors. This conclusion is supported by the GSEA results (Figure 4D). The relative expression of specific genes in the longevity regulation pathway was shown in Figure 4E.

### 3.5 The Delay in the Avoidance Behavior of *C. elegans* Induced by FAEW Is Linked to H4K8ac Down‐Regulation

**FIGURE 5:**
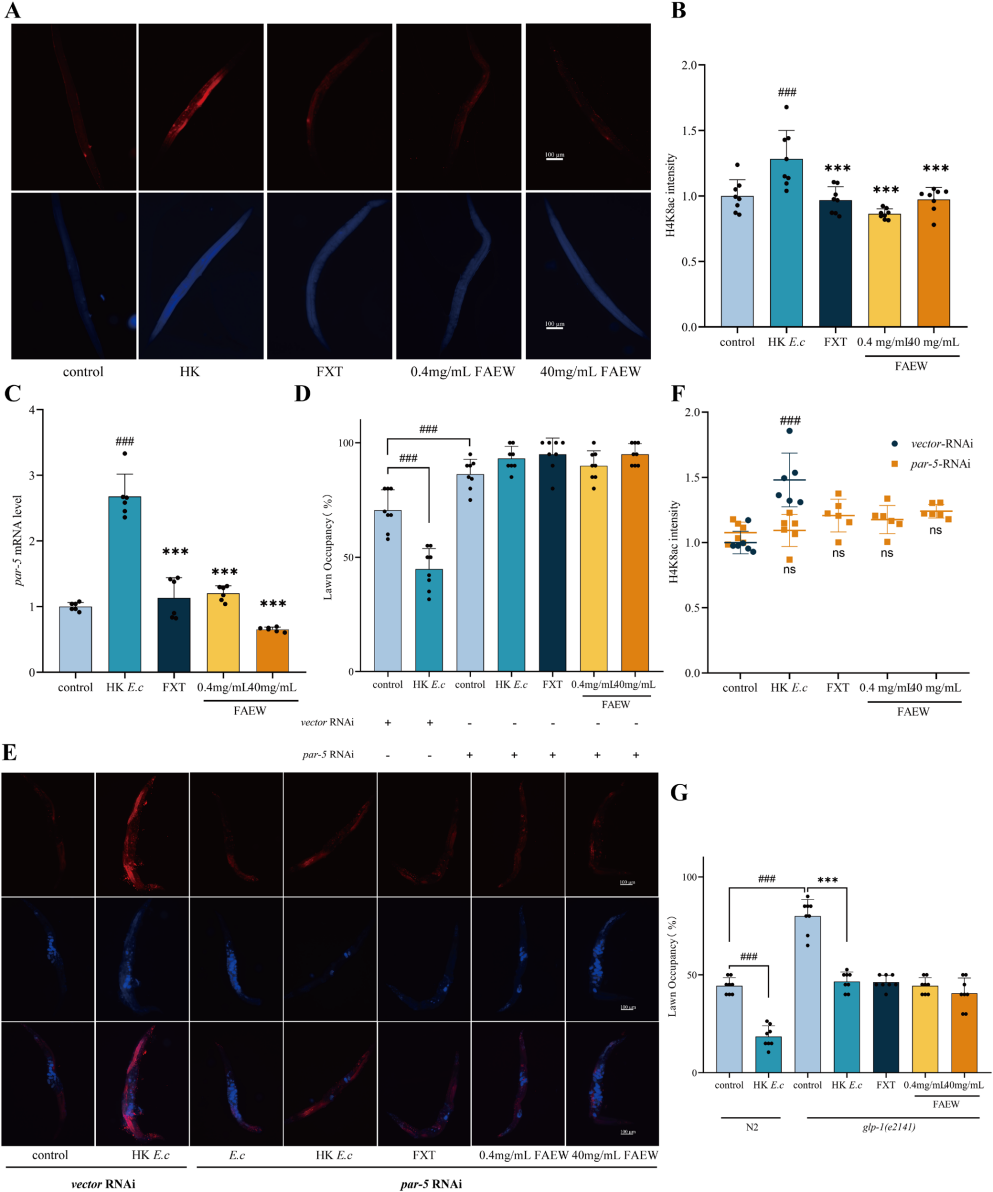
FAEW delays avoidance behavior by downregulating H4K8ac in the germline. (A) Representative microscopic images of wild‐type N2 animals stained with anti‐H4K8ac antibody. Scale bar, 100 μm. (B) Quantitative analysis of (A). The height of the column represents fold changes (*n* = 8). (C) qRT‐PCR for the *par‐5* gene in wild‐type N2 animals after FAEW and FXT administration based on HK *E. c* intervention (*n* = 6). (D) Lawn occupancy of *P. a* in animals with wild‐type N2 after FAEW and FXT administration upon the treatment of *par‐5* RNAi (*n* = 20). (E) Representative microscopic images of wild‐type N2 animals upon the treatment of *par‐5* RNAi stained with anti‐H4K8ac antibody. Scale bar, 100 μm. (F) Quantitative analysis of (D). The height of the column represents fold changes (*n* = 6). (G) Lawn occupancy of *P. a* in animals with wild‐type N2 and *glp‐1 (e2141)* mutants after FAEW and FXT administration based on HK *E. c* intervention (*n* = 20). In B‐C, *Comparison with HK *E. c*; #Comparison with control. In (D and E), #Comparison with *vector* RNAi‐*E. c*. In (G), *Comparison with *glp‐1 (e2141) E. c*; #Comparison with N2 *E. c*. ***, ###*p* < 0.001.

The research team had previously identified elevated levels of acetylated H4K8ac in *C. elegans* following *P. a* infection, a phenomenon associated with the regulation of avoidance behavior. Increased H4K8ac levels were observed irrespective of luminal distension induced by *P. a* infection for 24 h or normal feeding in constipated *aex‐5* and *eat‐2* mutants, indicating that H4K8ac elevation results from intestinal distension, suggesting gut‐germline‐neural signaling mediated pathogen avoidance behavior [25]. To understand the impact of FAEW on the H4K8ac levels in the germline, immunohistochemistry was employed. As shown in Figure 5A,B, HK *E. c* feeding induced H4K8ac expression with intensive red fluorescence, while both the positive control and FAEW can downregulate H4K8ac with weakened fluorescence.

Previous Co‐IP experiments have proved that the 14–3‐3 chaperone protein PAR‐5 can bind to H4K8ac and affect avoidance behavior. Silencing of the *par‐5* gene leads to reduced H4K8ac and delayed avoidance of *P. a*. In our study, we found that FAEW treatment decreased the expression of *par‐5* mRNA levels induced by HK *E. c* (Figure 5C). To further prove the effect, we observed the pathogen avoidance behavior of FAEW upon the treatment of *par‐5* RNAi. As shown in Figure 5D, FAEW had no effect on the delayed avoidance behavior conferred by gene silencing of *par‐5*. Similarly, FAEW did not affect H4K8ac levels in *par‐5* RNAi animals (Figure 5E,F). Together with the findings mentioned above (Figure 2B), it is inferred that FAEW delayed the adverse response of *C. elegans* to *P. a* following HK *E. c* feeding, which is correlated with enhanced intestinal lumen expansion, resulting in subsequent changes in *par‐5*‐mediated histone acetylation H4K8ac in the germline.

Furthermore, the *glp‐1* mutants, incapable of germline development due to the meiosis process, do not exhibit expression of H4K8ac. In this study, avoidance experiments using the *glp‐1* mutants (Figure 5D) showed that the effects of FAEW did not significantly impact the delayed avoidance caused by *glp‐1*. Thus, avoidance behavior resulting from intestinal lumen distension correlates with H4K8ac levels in the germline, and the potential mechanism underlying the inhibition of *C. elegans* avoidance behavior by FAEW may be linked to the amelioration of H4K8ac down‐regulation induced by intestinal lumen distension.

### 3.6 FAEW Modulates Avoidance Behavior in *C. elegans* Through Activating DAF‐16

**FIGURE 6:**
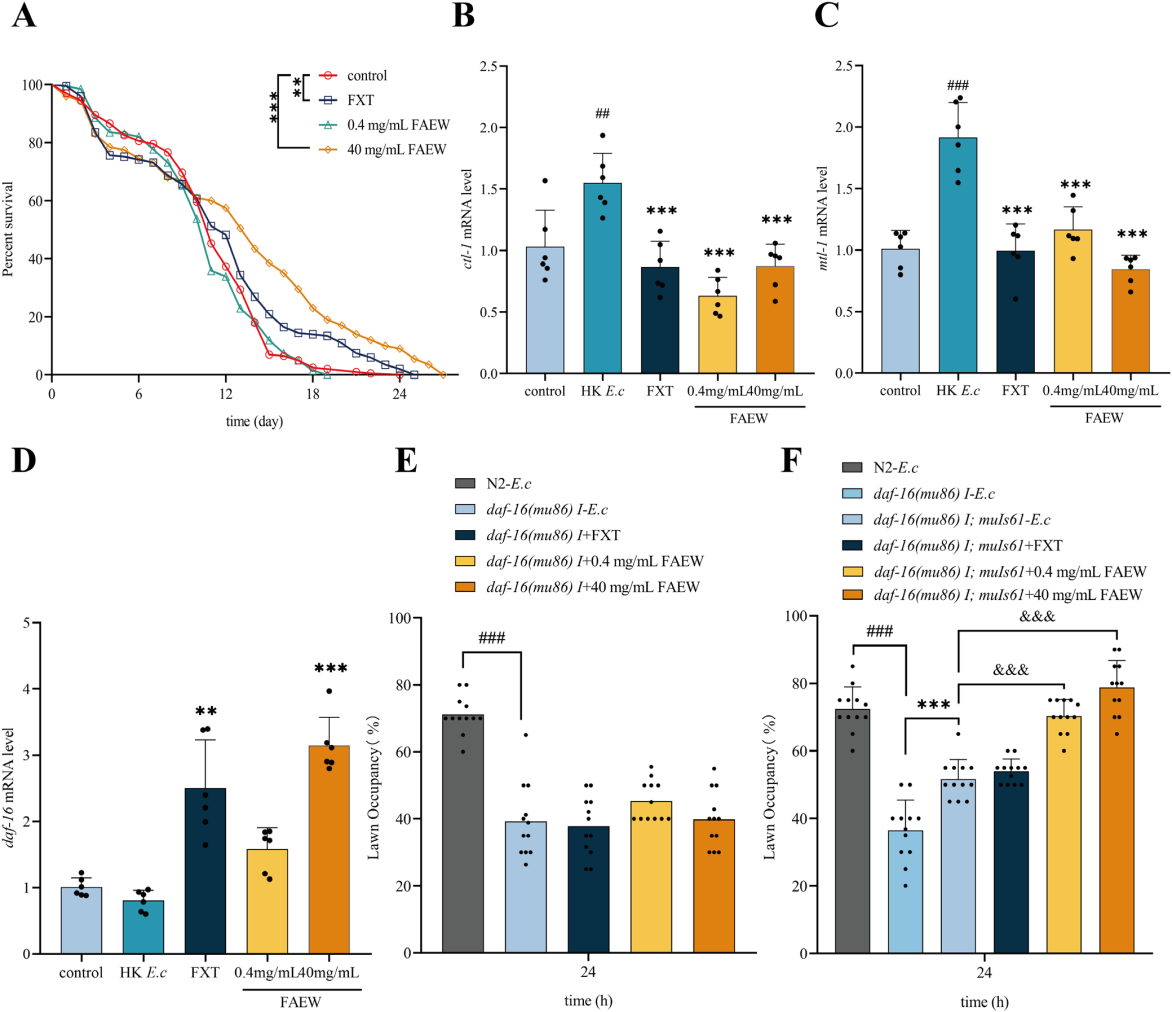
FAEW Modulates Avoidance Behavior in *C. elegans* Through Activating DAF‐16. (A) Lifespan assay of wild‐type N2 (WT) animals exposed to *E. c* following FAEW and FXT interventions (*n* = 50). (B–D) qRT‐PCR for the *ctl‐1, mtl‐1*, and *daf‐16* genes in wild‐type N2 animals after FAEW and FXT administration based on HK *E. c* intervention. (E) Lawn occupancy of *P. a* in animals with wild‐type N2 and *daf‐16(mu86) I*. mutants after FAEW and FXT administration based on HK *E. c* intervention (*n* = 20). (F) Lawn occupancy of *P. a* in animals with wild‐type N2 and *daf‐16 rescued animals daf‐16(mu86) I; muIs61* after FAEW and FXT administration based on HK *E. c* intervention (*n* = 20). In (A), *Comparison with control. In (B–D), #Comparison with control; *Comparison with HK *E.c*. In (E and F), *: Comparison with *daf‐16(mu86) I‐E. c*; #Comparison with N2‐*E. c*; &Comparison with *daf‐16(mu86) I; muIs61*‐*E. c*. **, ##*p* < 0.01; ***, ###, &&&*p* < 0.001.

It has been established that both the *par‐5* gene and the *glp‐1* signaling pathway in *C. elegans* are associated with increased lifespan [26, 27]. To confirm the effect of FAEW on aging and the potential signaling pathway suggested in the RNA sequencing data, firstly, we observed the effect of FAEW on lifespan, as shown in Figure 6A; FAEW significantly extended the lifespan of wild‐type animals. Secondly, a qRT‐PCR experiment was performed to validate the RNA sequencing data by taking some aging‐related genes; the results show the consistency between the two‐experiment methods of qRT‐PCR and RNA sequencing Figure 6B–D.

Numerous signaling pathways, including the insulin/IGF‐1 signaling pathway, mTOR signaling, AMPK pathway, JNK pathway, and germline signaling, have been identified as playing a role in the processes of aging and longevity [28, 29, 30, 31, 32]. DAF‐16/FOXO, serving as a pivotal transcription factor, possesses the capability to integrate diverse signals emanating from these pathways [33]. This integration allows for the modulation of aging and longevity by shuttling between the cytoplasm and nucleus. To confirm whether the effect of FAEW on avoidance behavior is through activating DAF‐16, we selected *daf‐16* mutants and the rescued animals to test the avoidance behavior of FAEW. As shown in Figure 6E,F, *daf‐16* mutants exhibit earlier avoidance behavior than wild‐type, while the delayed avoidance behavior’ effect of FAEW was affected in *daf‐16* mutants, showing no difference compared with *daf‐16* mutants but restored in *daf‐16* rescued animals. Taken together, these results indicate that FAEW regulates avoidance behavior of *C. elegans* through activating DAF‐16.

## 4 Discussion

*Caenorhabditis elegans* is a type of worm that lives in soil. Since the 1960s, scientists have used it as a model organism for biological research [34]. From a proteomic point of view, there are many similarities between nematodes, mammals, and rodents [35], with at least 83% of the *C. elegans* proteome having human homologues and at least 84% of the proteome likely to have mouse orthologues. Furthermore, the primary mechanisms involved in the biosynthesis and release of neurotransmitters, as well as the receptors that facilitate these processes, are found to be conserved in *C. elegans* . This species employs the process of exocytosis to release small‐molecule neurotransmitters, including glutamate (Glu), gamma‐aminobutyric acid (GABA), dopamine (DA), serotonin (5‐HT), and acetylcholine (ACh) [36]. The mechanisms of neurotransmission are similar to those found in mammals. The nervous system of *C. elegans* is structurally simple, functional, and easy to understand, containing only 302 neurons and about 7000 synapses, but its physiological functions and behavioral phenotypes are complex and diverse, and nearly complete neural connectivity has been mapped [37, 38]. *C. elegans* is the optimal model for investigating the relationship between the nervous system and behavior.

In recent years, it has been revealed that the gut –neural axis plays a crucial role in the occurrence of avoidance behavior in *C. elegans* . Lee et al. discovered that the neuropeptide INS‐11 secreted in the intestinal tract of *C. elegans* can reversibly regulate the avoidance behavior of *C. elegans* to aversive stimuli [39]. This mechanism is associated with the activation of the upstream transcription factor HLH‐30 and the p38 MAPK signaling pathway. Our group's preliminary studies have revealed that the intestine‐germline‐ural axis regulates the avoidance behavior of *C. elegans* [25]. Expansion of the intestinal lumen induced by *P. a* leads to acetylation of histone H4 lysine 8 (H4K8ac) in the germline of *C. elegans* , affecting its avoidance behavior towards pathogens. Furthermore, the occurrence of H4K8ac requires the participation of the 14–3‐3 protein family member PAR‐5. Worms with *glp‐1* mutations, due to meiosis processes, exhibit underdeveloped germline and lack detectable H4K8ac. Taken together, these results emphasize the crucial role of H4K8ac in the germline, not only in transmitting aversion to pathogens across generations but also in controlling avoidance behavior through the intestine‐neural axis, revealing key clues to the pathogenic process.

In this study, we observed that *Free and easy wander* significantly improved the intestinal expansion in *C. elegans* , downregulated histone acetylation H4K8ac, and decreased the expression of *par‐5*. The effect of FAEW on improving the avoidance behavior of *C. elegans* showed no significant difference compared to the *par‐5* silenced worms. This suggests that the molecular mechanism by which *Free and easy wander* improves the avoidance behavior of *C. elegans* is related to the activation of *par‐5*, highlighting that *Free and easy wander*, by targeting the intestine and modulating the intestine‐germline axis, improves intestinal expansion‐dependent avoidance behavior through the downregulation of H4K8ac level in the germline.

PTSD can pose challenges for individuals who have experienced or witnessed traumatic events. Growing evidence indicates a correlation between PTSD and the manifestation of accelerated aging [40, 41]. Drawing parallels, temperature‐sensitive *glp‐1* mutants, characterized by the absence of germline, exhibit an extended lifespan under non‐permissive temperatures [42]. Specifically, in *glp‐1* mutants, autophagy and LIPL‐4 function interdependently to modulate the aging process of germline‐defective *C. elegans* , thereby prolonging lifespan through the maintenance of lipid homeostasis [43]. Additionally, it has been demonstrated that *par‐5* can regulate lifespan and interact with histone deacetylase SIR‐2.1 and DAF‐16/FOXO [44]. Considering the regulatory role of FAEW in the avoidance behavior of *C. elegans* , upregulation of H4K8ac in the germline is involved, and *par‐5* gene expression is affected, lifespan‐related signaling pathways might be an underlying mechanism. In our investigation, RNA‐sequence analysis was performed to explore potential signaling pathways. The results, in conjunction with the lifespan assay, suggest that aging is intricately involved in FAEW's impact on avoidance behavior, thereby indicating that FAEW may regulate avoidance behavior in a lifespan‐dependent manner.

In *C. elegans* , DAF‐16 serves as a pivotal pro‐longevity transcription factor that modulates the Insulin/IGF‐1 signaling pathway. When exposed to various forms of stress, DAF‐16 translocates from the cytoplasm to the nucleus, where it orchestrates gene expression programs leading to an extension of lifespan [45, 46]. In our investigation, the delay in avoidance behavior induced by FAEW was absent in *daf‐16* mutants but restored to the original level in *daf‐16* rescued animals. This suggests that FAEW regulates avoidance behavior through *daf‐16*. Consequently, the impact of FAEW on avoidance behavior is contingent on lifespan and is mediated through *daf‐16*.

It's reported that the reproductive system of *C. elegans* communicates with the intestine through lipophilic hormone signals, and the *kri‐1* gene in the intestine responds to this signal, promoting DAF‐16 nuclear localization [47]. This suggests that the relationship between intestinal lumen expansion and germline histone acetylation in *C. elegans* needs to be further investigated. Studies show that intestinal distension results in a relative decrease in lipid deposition in *C. elegans* [48], which may lead to nematode malnutrition and abnormal lipid metabolism. Similarly, we found changes in lipid metabolism‐related pathways when we functionally enriched for the differentially expressed gene KEGG. One of them, LIPL‐3, a lipolytic enzyme, is highly expressed in intestinal cells, and its main function is to catalyze the lipid hydrolysis reaction, which can influence the generation of lipid molecules. Together with free fatty acids, they can act as signaling molecules to affect the expression of insulin signaling pathways and genes related to lipid metabolism. The breakdown product of energy catabolism of lipids, acetyl‐CoA, provides the substrate required for the post‐translational modification of acetylated histones by acetyltransferases. We speculate that *lipl‐3* may regulate lipid metabolism and affect histone acetylation, which may be the underlying mechanism of our gut‐germline‐neural signaling.

## 5 Conclusions

In conclusion, our results suggest that *Free and Easy Wanderer* ameliorates intestinal bloating‐dependent avoidance behavior of *C. elegans* through gut‐germline‐neural Signaling.

## Conflicts of Interest

We the undersigned declare that this manuscript is original, has not been published before, and is not currently being considered for publication elsewhere. We confirm that the manuscript has been reviewed and approved by all named authors. We further confirm that all of us participated sufficiently in the work and agree to take public responsibility for its content. We declare that there are no conflicts of interest.

## Supporting information


Figure S1.

Figure S2.

Table S1.

Table S2.

## Data Availability

The data that support the findings of this study are available on request from the corresponding author. The data are not publicly available due to privacy or ethical restrictions.
