# Supplementary figures and images for: Free and Easy Wanderer Ameliorates Intestinal Bloating‐Dependent Avoidance Behavior of Caenorhabditis elegans Through Gut‐Germline‐Neural Signaling
Source: CNS Neurosci Ther. 2025 Feb 26;31(2):e70291. doi: 10.1111/cns.70291 (PMC11862825; doi:10.1111/cns.70291)

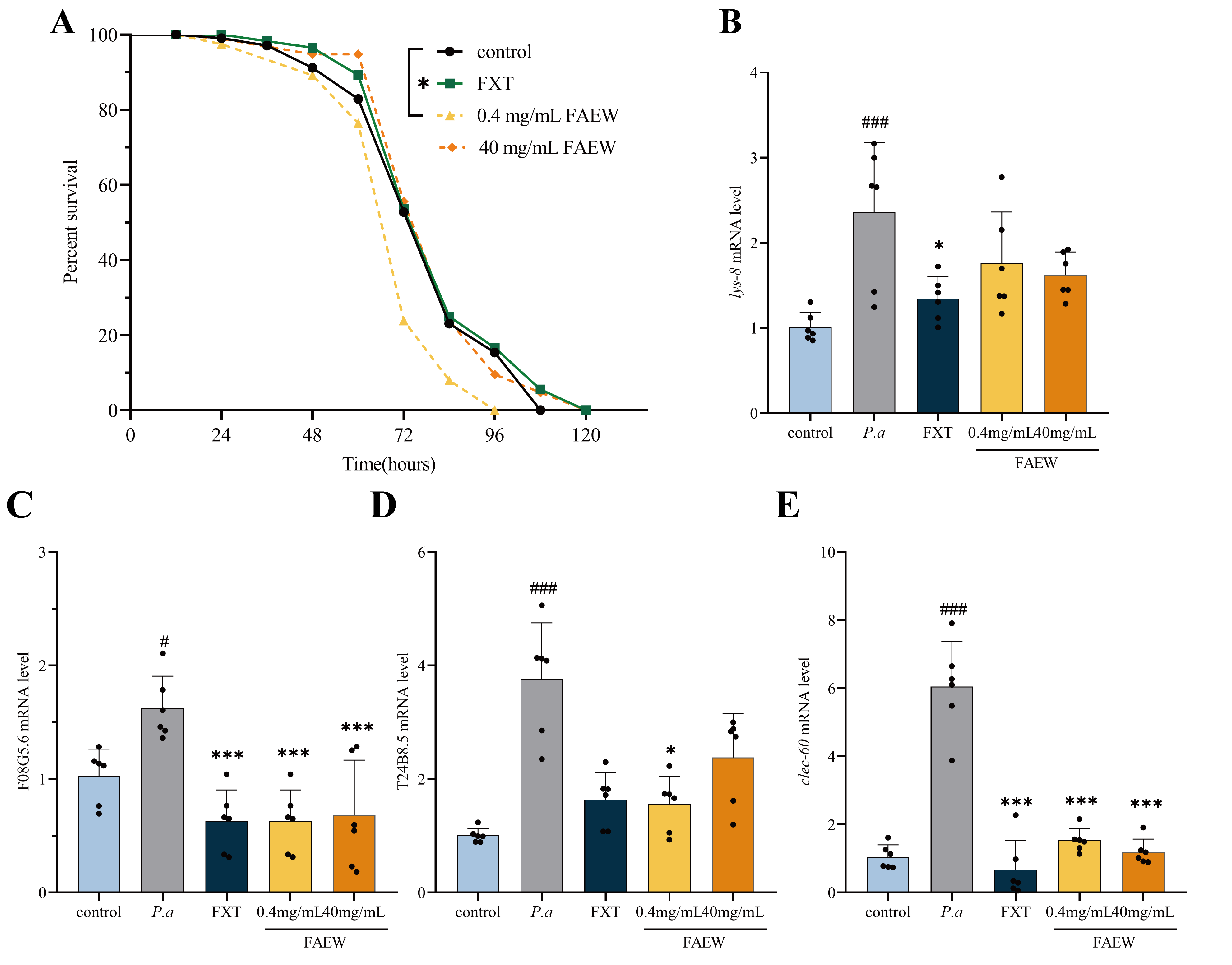

Supplement: Supplementary file 1 — Figure S1. [file CNS-31-e70291-s001.png]
